# Army Medical Examinations

**Published:** 1896-07

**Authors:** 


### ARMY MEDICAL EXAMINATIONS.

### VACANCIES IN THE MEDICAL CORPS OF THE U. S. ARMY.

THERE are three vacancies in the Medical Corps of the U. S. Army, and it is expected that three more will occur during this year. An army medical board will meet in Washington early in October for the examination of candidates. The requirements for admission are as follows:

Permission to appear before the Board is obtained by letter to the Secretary of War, which must be in the handwriting of the applicant, giving the date and place of his birth and the place and state of which he is a permanent resident, and inclosing certificates, based on personal acquaintance, from at least two reputable persons as to his citizenship, character and habits. The candidate must be a citizen of the United States, between twenty-two and twenty-nine years old, of sound health and good character, and a graduate of some regular medical college, in evidence of which his diploma will be submitted to the board. The scope of the examination will include the morals, habits, physical and mental qualifications of the candidate, and his general aptitude for service ; and the board will report unfavorably should it have a reasonable doubt of his efficiency in any of these particulars.

The physical examination comes first in order, and must be thorough. Candidates who fall below sixty-four inches in height will be rejected. Each candidate will also be required to certify "that he labors under no mental or physical infirmity or disability which can interfere with the efficient discharge of any duty which may be required." Errors of refraction, when not excessive, and not accompanied by ocular disease, and when correctible by appropriate glasses, are not causes for rejection.

The mental examinations are conducted by both written and oral questions, upon—

I. Elementary branches of a common school education, including arithmetic, the history and geography of the United States, physics, ancient and modern history, and general literature. Candidates claiming especial knowledge of the higher mathematics, ancient or modern languages, drawing, analytical chemistry or branches of natural science, will be examined in those subjects as accomplishments, and will receive due credit therefor according to their proficiency.

II. Professional branches, including anatomy, physiology, chemistry, hygiene, pathology and bacteriology, therapeutics and materia medica, surgery, practice of medicine, obstetrics and the diseases of women and children.

Examinations will also be conducted at the bedside in clinical medicine and surgery, and operations and demonstrations will be made by the candidates upon the cadaver.

Hospital training and practical experience in the practice of medicine, surgery and obstetrics are essential to candidates seeking admission to the medical corps of the army, who will be expected to present evidence that they have had at least one year's hospital experience or the equivalent of this in practice.

To save unnecessary expense to candidates, those who desire it may have a preliminary physical examination and a mental examination in the "elementary branches of a common school education," by a medical officer of the army, stationed most conveniently for this purpose, who will act under instructions from the medical examining board.

